# Susceptibility of *Bemisia tabaci* MEAM1 (Hemiptera: Aleyrodidae) to Imidacloprid, Thiamethoxam, Dinotefuran and Flupyradifurone in South Florida

**DOI:** 10.3390/insects7040057

**Published:** 2016-10-20

**Authors:** Hugh A. Smith, Curtis A. Nagle, Charles A. MacVean, Cindy L. McKenzie

**Affiliations:** 1 Gulf Coast Research and Education Center, University of Florida, Wimauma, FL 33598, USA; cnagle@ufl.edu; 2 School of Sciences, Saint Francis University, Loretto, PA 15940, USA; cmacvean@francis.edu; 3 USDA-ARS, Fort Pierce, FL 34945, USA; cindy.mckenzie@ars.usda.gov

**Keywords:** insecticide resistance, neonicotinoid insecticides, butenolide insecticides

## Abstract

Populations of *Bemisa tabaci* MEAM1 were established from nineteen locations in south Florida, primarily from commercial tomato fields, and were tested using a cotton leaf petiole systemic uptake method for susceptibility to the nicotinic acetylcholine agonist insecticides imidacloprid, thiamethoxam, dinotefuran and flupyradifurone. Eleven populations produced LC_50_s for one or more chemicals that were not significantly different from the susceptible laboratory colony based on overlapping fiducial limits, indicating some degree of susceptibility. LC_50_s more than a 100-fold the laboratory colony were measured in at least one population for each material tested, indicating tolerance. LC_50_s (ppm) from field populations ranged from 0.901–24.952 for imidacloprid, 0.965–24.430 for thiamethoxam, 0.043–3.350 for dinotefuran and 0.011–1.471 for flupyradifurone. Based on overlapping fiducial limits, there were no significant differences in relative mean potency estimates for flupyradifurone and dinotefuran in relation to imidacloprid and thiamethoxam.

## 1. Introduction

The sweetpotato whitefly, *Bemisia tabaci* (Gennadius) Middle East Asia Minor 1 (MEAM1) (Hemiptera: Aleyrodidae), formerly known as *B. tabaci* biotype B, is a globally important pest of agronomic, horticultural and ornamental crops [1]. *Bemisia tabaci* debilitates plants through removal of sap and contaminates crops by producing honeydew, a substrate for sooty mold. The whitefly causes significant crop losses globally by transmitting >150 species of viruses, and by inducing plant disorders such as silverleafing of squash and irregular ripening of tomato [2]. *Bemisia tabaci* is the primary pest of tomato (*Solanum lycopersicum* L.) in Florida [3]. It transmits *Tomato yellow leaf curl virus* (TYLCV), a geminivirus that can cause complete loss of crops [4]. The earlier the crop is infected with TYLCV, the greater the impact is on yield [5]. Florida is the foremost producer of fresh market tomatoes in the United States, with over 33,000 acres harvested in 2014 at a value of $437 million [6]. The MEAM1 species of *B. tabaci* first became established in Florida in the late 1980s, and TYLCV was first detected in 1997 [7]. *Bemisa tabaci* and TYLCV are managed in Florida tomato with a combination of clean culture (destruction of harvested tomato fields as the primary sources of inoculum), deployment of virus tolerant tomato varieties, use of metalized plastic mulches to repel the vector, and intensive insecticide use [8].

Since the registration of imidacloprid (Admire Pro, Bayer Crop Science, Raleigh, NC, USA) for Florida tomato in 1993, the neonicotinoid insecticides have played a major role in chemical control of *B. tabaci* and geminiviruses in the state. Neonicotinoids are agonists of nicotinic acetylcholine receptors in insects [9]. Tomato seedlings are typically treated with imidacloprid or thiamethoxam (Platinum, Syngenta Corporation, Greensboro, NC, USA) in the nursery. Seedlings are usually treated again at-planting with either imidacloprid, thiamethoxam or dinotefuran (Venom, Valent BioSciences, Libertyville, IL, USA), each of which may be subsequently applied through the drip irrigation or foliarly [10]. The University of Florida recommends that the use of neonicotinoid insecticides be confined to the first five week treatment window after planting of tomato to avoid the development of resistance in whitefly populations [10,11]. *Bemisia tabaci* has developed tolerance to carbamate, organophosphate, and pyrethroid insecticides in different regions of the globe, and to newer insecticides, including pymetrozine and pyriproxifen [12,13,14]. *Bemisia tabaci* resistance to imidacloprid was first documented in the Almeria region of southern Spain in the middle of 1990s [15]. Resistance to imidacloprid, thiamethoxam and other neonicotinoid insecticides has been documented among *B. tabaci* populations in several parts of the world [9] including the southwestern USA [16,17] and Florida [18,19]. The Mediterranean (MED) group of *Bemisia tabaci* (commonly known as biotype Q) was detected in Florida in 2005 in ornamental nurseries [20]. *Bemisia tabaci* MED is characterized by the ability to develop high levels of resistance to pyriproxifen and some neonicotinoid insecticides [21,22]. The widespread establishment of *B. tabaci* MED in Florida field crops would require fundamental changes in approaches to managing the pest. Surveys of *B. tabaci* in tomato and other horticultural crops are needed to detect any change in the *B. tabaci* species composition.

Schuster et al. [18] found fluctuations in average levels of resistance to imidacloprid and thiamethoxam among whitefly populations in Florida between 2000–2008, with resistance ratios (RRs) peaking at 26.3 for imidacloprid and at 24.7 for thiamethoxam. Concerns regarding neonicotinoids have been compounded in recent years by the perception that neonicotinoids may have a negative impact on pollinators [23]. Flupyradifurone (Sivanto 200 SL, Bayer Crop Science, Raleigh, NC, USA) has the same mode of action as the neonicotinoids but belongs to the butenolide group of insecticides and has a positive pollinator safety profile [24]. Flupyradifurone has demonstrated efficacy in the control of *B. tabaci* and TYLCV in greenhouse and field studies in Florida [11,25,26]. Flupyradifurone was registered for use on Florida tomato Feb 2015.

In order to assess the susceptibility of *B. tabaci* to four nicotinic acetylcholine receptor agonist insecticides, the F2, F3 or F4 of populations collected from the field were tested using a systemic cotton petiole assay in the laboratory and compared to a laboratory colony of *B.tabaci* that has not been exposed to insecticides since the late 1980s [19]. *Bemisia tabaci* is the only whitefly affecting tomato production in Florida and so is easily identified. Most populations were collected from late season commercial (>20 ha) tomato fields treated repeatedly with conventional insecticides for management of whiteflies and other pests. One population was collected from a commercial squash (*Cucurbita pepo* Mill) field. Information was not collected on the specific insecticide history of each field. Two populations were collected from a common wild whitefly host, *Emilia fosbergii* Nicolson, in order to determine if susceptibility was higher in general on host plants that had not been treated with insecticides. In addition to determining the relative susceptibility of field populations of *B. tabaci* to three insecticides that have been in common use for several years (imidacloprid, thiamethoxam, and dinotefuran), our objective was to gather baseline information on the susceptibility of *B. tabaci* to flupyradifurone, which was not commercially available at the time the populations were collected.

## 2. Materials and Methods

Studies were completed at the University of Florida Gulf Coast Research and Education Center (GCREC), Balm, FL (27°45.599′ N, 82°13.446′ W) between October 2013 and February 2015 to evaluate the susceptibility of field populations of *B. tabaci* MEAM1 to imidacloprid, thiamethoxam, dinotefuran and flupyradifurone.

### 2.1. Collection of Field Colonies

Nineteen *Bemisia tabaci* populations were collected October 2013–December 2014 from Hendry, Hillsborough, Indian River, Manatee, and Miami-Dade counties. To establish colonies of whiteflies from field populations, twelve 6-week old “Lanai” tomato plants in 3.78 liter pots were placed among crop plants in 15 tomato fields and 1 squash field. Late season fields were chosen to increase the likelihood that whitefly populations had been exposed to insecticides. The “Lanai” tomato plants were left for 3–4 days in the field to allow whitefly females the opportunity to find and oviposit on them. Exposed “Lanai” tomato plants were then returned to GCREC and placed in a growth room on an open bench with 3-week old cotton (*Gossypium hirsutum* L., cv. Delta Pine 143 B2RF) plants, grown in 10-cm pots. Cotton is the standard plant used for whitefly resistance bioassays [27]. Two whitefly populations were established from infested *E. fosbergii* plants adjacent to tomato fields. All whitefly populations were reared in growth rooms with 12:12 h light: dark photoperiod provided by fluorescent lights and a temperature range of 75–85 °F (24–29.5 °C). Cotton plants were added weekly to growth rooms, and as host plants senesced, whitefly adults from the field and their adult progeny moved over to the cotton. Whitefly populations were allowed to build on cotton for 6–12 weeks with the result that the F2–F4 generations were tested (Table 1). LC_50_s of field populations of *B. tabaci* were compared to LC_50_s of a susceptible colony that has been in culture on cotton at GCREC since the late 1980s [19].

### 2.2. Bioassay Technique

**Figure 1:**
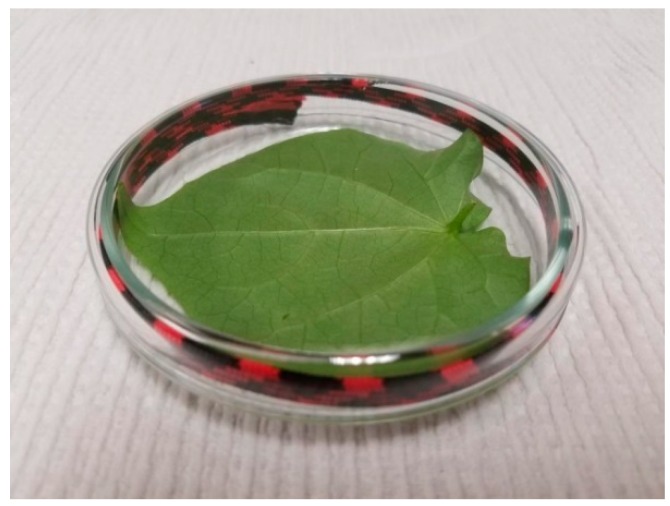
This figure illustrates a treated cotton leaf enclosed in a Petri dish. Whitefly adults were confined within the Petri dish on the treated leaf for 72 h, at which time mortality data were collected.

Insecticide solutions were prepared by serial dilution from commercially formulated products. The products evaluated were imidacloprid (Admire Pro, Bayer Crop Science, Raleigh, NC, USA); flupyradifurone (Sivanto 200 SL, Bayer Crop Science); thiamethoxam (Platinum 75SG, Syngenta Corporation, Greensboro, NC, USA), and dinotefuran (Venom 70SG, Valent BioSciences Corporation, Libertyville, IL, USA). The treatments were based on those used by Schuster et al. (2010) [18] and consisted of 0, 0.1, 1.0, 10, 75.0, and 300 ppm active ingredient in 50 ml of deionized water. A cotton leaf petiole systemic uptake method was used to expose whiteflies to treatments [18]. Cotton plants were grown in growth rooms with a 14:10 h light: dark photoperiod. The leaves were cut from the plants and their petioles were immersed into a 50 mL Erlenmeyer flask containing a preparation of insecticide or pure water. Usually the first leaves above the cotyledons were used because they fit well inside the Petri dish where whiteflies were confined on the treated leaf for the duration of the test (Figure 1).

The leaves were left for 24 h under natural and fluorescent light at a temperature range of 67–76 °F (19.4–24.4 °C). On the following morning, the leaves were removed from their flasks; the petioles were cut off at about 0.6 cm from the leaf blade and placed into glass 100 × 15 mm diameter Petri dishes (KIMAX no. 53062/53064-10015, Fisher Scientific, Waltham, MA, USA). 10 to 15 adult whiteflies of mixed age and gender were aspirated into a 2 mL glass Dropping Pipette (American Educational Products no. S18765, Fort Collins, CO, USA) fitted with a foam rubber filter. The specific number of whiteflies per Petri dish was confirmed at 72 h, when final mortality data were collected. Each group of adults in a pipette was an experimental unit and was placed into a freezer (−12 °C) for 1 min. Immobilized adults were then tapped out of the pipette onto the cotton leaf inside the bottom half of a Petri dish and covered with the top half. Paracord (Lehigh Consumer Products LLC, Macungie, PA, USA) was placed between the halves of the dishes to prevent whiteflies from escaping while allowing air circulation. Each Petri dish represented a replication of a treatment, and each treatment was replicated four times. Petri dishes were stacked in four layers (insecticides) of six dishes (concentrations) within an Office Depot Brand 39 × 27 × 14 cm clear plastic storage box with four water saturated cotton rolls arranged around the bottom of the box to maintain high relative humidity. Each test consisted of one test population and required 1 week to execute. Each population was tested 1–3 times. Populations tested more than once were tested over consecutive weeks.

### 2.3. Bioassay Sampling

Response of the whiteflies to treatments was assessed 72 h after introduction to the Petri dishes. Adult whiteflies were observed using a dissecting microscope and recorded as “live” if they appeared normal, “moribund” if there was movement which seemed abnormal, or “dead” if no movement was detected. Whiteflies exhibiting ambiguous behavior were prodded with a probe to confirm status.

### 2.4. Statistical Analysis

Probit analysis was performed using IBM SPSS Statistics (ver. 22) software (IBM Corporation, Armonk, NY, USA) [28]. Populations (locations) were analyzed separately, generating LC_50_s for all chemicals for each population. A pooled analysis was also run for all populations together, generating overall LC_50_s for each chemical. Only whiteflies recorded as “dead” at 72 h were included in the “responding” group for the analysis. LC_50_s for each population (with 95% fiducial limits) were calculated for each insecticide. Relative mean potency (RMP) was determined for all possible chemical pairs in each population as the ratio of LC_50_ chemical 1/ LC_50_ chemical 2. An RMP allows a comparison of any two field LC_50_s for efficacy.

### 2.5. Whitefly Biotype Determination

Whitefly DNA extraction, amplification, gel visualization.

Twelve whiteflies from each population were used for species determination following the protocol developed by Shatters et al [29]. DNA was extracted from individual whiteflies by placing a single whitefly in a 1.5 mL Eppendorf tube, adding 50 µL of DNA lysis buffer and grinding with a pestle. The pestle was rinsed with an additional 50 µL of DNA lysis buffer and collected in the same tube. Tubes were placed in a metal boiling rack and boiled at 95 °C for 5 min then placed directly in ice for 5 min. Tubes were then centrifuged at 8000× *g* for 30 s and the supernatant (crude DNA lysate) was transferred to another tube and stored at −80 °C for future processing.

Species (biotype) specific PCR-primers designed by Shatters et al. [29] to recognize unique mtCOI gene regions within each of the MEAM1 (B), MED (Q), and New World species and to produce different sized products depending on the source of the isolated template DNA were used. Each mtCOI primer pair was chosen to amplify a species specific fragment of a different size with the MED, New World and MEAM1 species amplified fragments of 303 bp, 405 bp and 478 bp, respectively. By combining these primers and employing rapid PCR and electrophoretic techniques, biotype determination can be made within three hours for up to 96 samples at a time. The 30 µL final volume PCR reactions were run using a MJ Research PTC-200 Peltier thermal cycler (MJ Research Inc., Waltham, MA, USA). The temperature profile for the amplification of the mtCOI gene fragments was: (1) denaturation at 94 °C for 2 min; (2) 35 cycles of: 94 °C for 30 s, 64 °C for 1 min, and 72 °C for 1 min; and (3) final elongation at 72 °C for 10 min. PCR products were separated by gel electrophoresis using 1.5% agarose gels containing 50 µg per 100 mL gel of ethidium bromide.

## 3. Results

**Figure 2:**
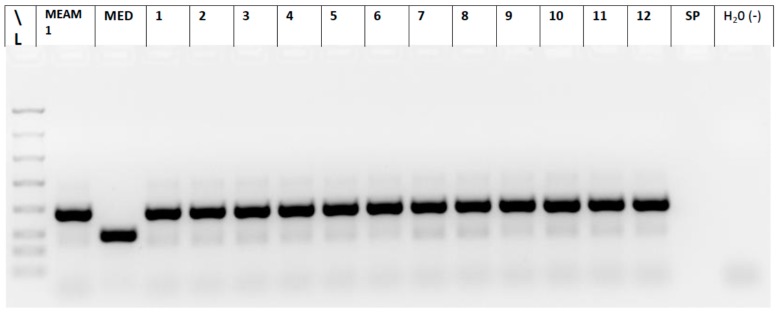
Amplification of *Bemisia tabaci* mtCOI with biotype specific primers to distinguish the B biotype (MEAM1) and Q biotype (MED) the IndianRiver-Squash population. Total DNA preparations were used as the template in 30 µL of PCR reactions containing two sets of biotype specific primers for the B (MEAM1) and Q (MED) biotypes. L, FlashGel^®^ (Lonza Rockland, Inc., Rockland, ME, USA) Dye and Marker, 100 bp to 4 kb, (+) B MEAM1: 478 bp; (+) Q MED: 303 bp; Lanes 12-1 to 12-12: whitefly sampled from squash; site 12: Vero Beach; SP: space; H_2_O (−): negative water control with primers only.

All populations of *B. tabaci* were confirmed to be MEAM1. Figure 2 shows the banding pattern produced by adult whitefly samples from the IndianRiver-Squash population. This gel is representative of the nineteen populations tested.

The lowest LC_50_s (ppm) measured for each insecticide were derived from the susceptible laboratory colony. The LC_50_s (and 95% fiducial limits) for the laboratory colony were 0.131 (0.018–0.457) for imidacloprid (Table 1), 0.139 (0.021–0.460) for thiamethoxam (Table 2), 0.026 (0.002–0.122) for dinotefuran (Table 3) and 0.010 (0.001–0.060) for flupyradifurone (Table 4). LC_50_s from field populations ranged from 0.901–24.95 for imidacloprid, 0.97–24.43 for thiamethoxam, 0.043–3.35 for dinotefuran and 0.011–1.47 for flupyradifurone.

Based on overlapping fiducial limits, LC_50_s were not significantly different from the laboratory colony for one or more chemicals in eleven populations (Table 1, Table 2, Table 3 and Table 4). LC_50_s for Hendry3 and Manatee4 were not significantly different from the laboratory colony for any of the four insecticides tested. In total, LC_50_s for imidacloprid from four populations were not significantly different from the laboratory LC_50_; LC_50_s for thiamethoxam from five populations were not significantly different from the laboratory colony; LC_50_s for flupyradifurone from nine populations were not significantly different from the laboratory colony; and LC_50_s for dinotefuran from ten populations were not significantly different from the laboratory colony. The population reared from *E. fosbergii* from Indian River County was largely susceptible to the insecticides tested while the population reared from *E. fosbergii* from Hillsborough County was mostly tolerant: the LC_50_s for imidacloprid, dinotefuran and flupyradifurone produced by the IndianRiver-Emilia population were not significantly different from the laboratory colony based on overlapping fiducial limits; the LC_50_s produced by the Hillsborough-Emilia population were from 15 (dinotefuran) to 41-fold (thiamethoxam) greater than the laboratory colony, with no overlap in fiducial limits.

Overall LC_50_s (and 95% fiducial limits) calculated from pooled field populations were 4.169 (3.449–5.020) for imidacloprid, 4.038 (3.347–4.854) for thiamethoxam, 0.422 (0.329–0.535) for dinotefuran and 0.252 (0.193–0.324) for flupyradifurone. Based on overlapping fiducial limits, LC_50_s for imidacloprid and thiamethoxam were not significantly different. LC_50_s for dinotefuran and flupyradifurone did not overlap, however the difference between the upper bound for flupyradifurone and the lower bound for dinotefuran was 0.005.

Flupyradifurone produced pooled (all field populations combined) relative mean potency estimates (and 95% confidence intervals) that were 16.5 (12.098–23.010) times greater than imidacloprid (i.e., imidacloprid’s LC_50_ is 16.5 times higher than flupyradifurone’s), 16.0 (11.752–22.212) times greater than thiamethoxam and 1.67 (1.255–2.244) times greater than dinotefuran. Pooled relative mean potency estimates for dinotefuran were 9.878 (7.388–13.475) times greater than imidacloprid and 9.567 (7.126–13.011) times greater than thiamethoxam. The relative mean potency estimate for thiamethoxam in relation to imidacloprid was 1.032 (0.798–1.337). Based on overlapping fiducial limits, there were no statistical differences in relative mean potency estimates for flupyradifurone and dinotefuran in relation to imidacloprid and thiamethoxam.

## 4. Discussion

Resistance monitoring is a key component of integrated management of *B. tabaci*, which incorporates cultural controls and host plant resistance to reduce overreliance on insecticides for management of *B. tabaci* and TYLCV [10,30]. Monitoring tolerance of *B. tabaci* to neonicotinoid insecticides and establishing baseline information for new insecticides such as flupyradifurone are essential for the sustainable stewardship of crucial crop protection tools. Imidacloprid was first registered for use in Florida vegetable production in 1993. Thiamethoxam became available in 2003, followed by dinotefuran in 2005. These insecticides are used year-round to manage *B. tabaci* on many horticultural and ornamental crops in addition to tomato, resulting in constant selection pressure. Unsprayed refuges, such as wild hosts, might offer escape from insecticide pressure. However LC_50_s from the population reared from *E. fosbergii* in Hillsborough County indicated that tolerance may be maintained among populations developing on wild hosts.

We found evidence of susceptibility to all insecticides tested among some populations: four populations (21%) produced LC_50_s for imidacloprid that were not significantly different from the laboratory colony based on overlapping fiducial limits; by the same criteria, 26% of the populations exhibited susceptibility to thiamethoxam, 47% exhibited susceptibility to flupyradifurone and 53% exhibited susceptibility to dinotefuran.

The range of LC_50_s for imidacloprid and thiamethoxam measured from field populations of *B. tabaci* was similar to the range reported by Schuster et al. [18] from field surveys carried out in south and central Florida in 2007. The greatest LC_50_s measured for imidacloprid and thiamethoxam that year were slightly higher than the highest measured in our survey (32.5 vs. 24.9 for imidacloprid and 31.1 vs. 24.4 for thiamethoxam). Increasing degrees of tolerance to imidacloprid and thiamethoxam were evident among the remaining populations in our assays, including Hendry1, Miami-Dade3 and Hillsborough5, which produced LC_50_s for imidacloprid and/or thiamethoxam that were more than 100-fold the LC_50_s for the laboratory colony. These results indicate that there continues to be considerable variability in the susceptibility of different populations of *B. tabaci* to imidacloprid and thiamethoxam in central and south Florida. In spite of the fact that imidacloprid and thiamethoxam have been used intensively in the region since 1993 and 2003, respectively, some populations of *B. tabaci* continue to be susceptible to these insecticides.

The susceptible colony LC_50_s for dinotefuran and flupyradifurone were an order of magnitude lower than for imidacloprid and thiamethoxam. This difference was also reflected in the range of LC_50_s from field populations for dinotefuran and flupyradifurone, which were an order of magnitude lower than for the other two insecticides. The highest LC_50_s for dinotefuran measured from a field population were an order of magnitude greater than those observed by Caballero et al. [19] from Florida whiteflies in 2010 (0.335 vs. 3.350 ppm). We used the same bioassay method as Caballero et al. [19]. Our results indicate that dinotefuran was consistently more toxic to *B. tabaci* than both imidacloprid and thiamethoxam. This is consistent with what has been observed among *B. tabaci* populations in the desert valleys of California and Arizona [16].

This report provides the first data on base-line susceptibility of *B. tabaci* to flupyradifurone in Florida. Flupyradifurone (Sivanto 200 SL) first became commercially available for use in Florida vegetables in 2015, shortly after the last populations in this study were collected from the field. Flupyradifurone possesses the same mode of action as the neonicotinoid insecticides, but like dinotefuran, is not detoxified by the microsomal monooxygenases, including the P450 gene CYP6CM1, that have been implicated in the development of resistance to imidacloprid and other neonicotinoid insecticides by *B. tabaci* [24,31,32]. Nauen et al. [31] demonstrated that flupyradifurone lacks metabolic cross-resistance to imidacloprid. Qiong et al. [33] did not find cross resistance between dinotefuran and imidacloprid, thiamethoxam or acetamiprid. In our study, there was not a clear separation between flupyradifurone and dinotefuran with regard to LC_50_ values and measures of relative mean potency. Four populations collected from commercial tomato fields produced LC_50_s for flupyradifurone between 55 and 79-fold the LC_50_ of the laboratory colony. This is indicative of some degree of tolerance even though the populations could not have been exposed to flupyradifurone. By contrast, only one population produced an LC_50_ greater than 48-fold the laboratory colony for dinotefuran: the highly tolerant Hendry1 population. This population produced an LC_50_ for dinotefuran that was 129-fold that of the laboratory colony. It also produced the highest LC_50_ measured for flupyradifurone, 147-fold that of the laboratory colony.

The five field populations that exhibited LC_50_s for flupyradifurone more than 54-fold the laboratory colony also exhibited the five highest LC_50_s for dinotefuran (Hendry1, Hillsborough1 and 5, Miami-Dade1 and 2). The three highest LC_50_s measured for imidacloprid and thiamethoxam were also in this group of five populations. While cross-resistance between flupyradifurone and neonicotinoids is not predicted [24], the populations we tested were in several cases generally susceptible to all four compounds or generally tolerant, when LC_50_s are compared to the susceptible laboratory colony.

Patterns of cross-resistance within the neonicotinoid group as well as between neonicotinoids and other insecticides are poorly understood. Cahill et al. [15] determined that resistance to organophosphate, carbamate, pyrethroid and cyclodiene insecticides did not confer resistance to imidacloprid in the B strain of *B. tabaci* collected from the Almeria region of southern Spain. Prabhaker et al. [16] found that imidacloprid-resistant *B. tabaci* from Guatemala were cross resistant to acetamiprid, dinotefuran and thiamethoxam, but that imidacloprid-resistant conspecifics from the southwestern USA remained susceptible to those insecticides. Feng et al. [34] observed cross resistance to imidacloprid and acetamiprid in thiamethoxam-resistant *B. tabaci* MEAM1 in China. By contrast Horowitz et al. [22], studying *B. tabaci* from cotton in Israel, determined that resistance to thiamethoxam did not confer resistance to acetamiprid, but that resistance to acetamiprid produced up to 500-fold resistance to thiamethoxam. Pymetrozine is a pyridine azomethine antifeedant insecticide that is unrelated to the neonicotinoids structurally and functionally. However resistance to pymetrozine and neonicotinoids in *B. tabaci* is primarily due to the same mechanism [35]. Evidence of cross resistance between neonicotinoids and pymetrozine [35] and the example of whiteflies exposed only to acetamiprid developing resistance to thiamethoxam [22] illustrate the potential of *B. tabaci* to develop tolerance to an active ingredient to which it has not been exposed. It is also important to keep in mind that whiteflies in our assays and in studies mentioned above have typically been exposed to a suite of insecticides applied to manage a complex of pests in addition to *B. tabaci*.

## 5. Conclusions

The results of our research demonstrate that populations of *B. tabaci* in central and south Florida exhibit considerable variability in their susceptibility to imidacloprid, thiamethoxam, dinotefuran and flupyradifurone. No population exhibited a high degree of susceptibility to one insecticide while demonstrating tolerance to others. This was particularly noteworthy with regard to flupyradifurone, given that no populations had been exposed to the compound in the field. Populations with the highest tolerance to flupyradifurone also demonstrated the highest tolerances for the other insecticides tested, indicating some degree of cross resistance. The results of this study will contribute to resistance management guidelines for *B. tabaci* in tomato and other vegetables.

## Figures and Tables

**Table 1:** Dose response to imidacloprid and resistance ratios of *Bemisia tabaci* adults, reared from Florida field-collected populations, as measured by mortality (LC_50_) with a cut cotton leaf and petiole systemic uptake bioassay in the laboratory.

Population Code ^1^	Mortality			Resistance Ratio ^4^
	No. Tested ^2^	Slope ± SE	LC_50_ (95% CL) ^3^	
Laboratory	492	0.564 ± 0.054	0.131 (0.018–0.457)	-
Hendry3	465	0.828 ± 0.058	0.901 (0.411–1.689)	6.88
Manatee1	412	0.481 ± 0.040	0.995 (0.263–2.727)	7.60
Manatee4	234	0.457 ± 0.047	1.349 (0.245–5.067)	10.30
Hillsborough3	449	0.522 ± 0.041	1.584 (0.502–3.881)	12.09
IndianRiver-Emilia	292	0.570 ± 0.045	1.601 (0.450–4.651)	12.22
Hendry2	371	0.705 ± 0.063	1.721 (0.670–3.516)	13.14
Manatee3	570	0.620 ± 0.033	2.055 (0.947–4.168)	15.69
Hillsborough-Emilia	542	0.641 ± 0.032	2.408 (1.249–4.431)	18.38
Manatee2	491	0.502 ± 0.031	2.629 (1.087–5.913)	20.07
IndianRiver-Squash	795	0.566 ± 0.026	4.112 (2.133–7.690)	31.39
Hillsborough4	235	0.460 ± 0.045	4.677 (1.118–16.650)	35.70
Hillsborough1	322	1.216 ± 0.072	5.326 (3.143–8.768)	40.66
Miami-Dade1	526	0.812 ± 0.047	9.098 (5.543–14.905)	69.45
IndianRiver1	373	0.707 ± 0.057	9.406 (4.700–18.968)	71.80
Hillsborough2	485	0.821 ± 0.052	10.590 (5.779–19.783)	80.84
Miami-Dade3	213	0.505 ± 0.045	11.994 (3.166–46.568)	91.56
Hendry1	581	0.749 ± 0.043	12.919 (8.089–20.894)	98.62
Hillsborough5	228	0.477 ± 0.044	21.524 (7.263–70.000)	164.31
Miami-Dade2	363	0.530 ± 0.052	24.952 (11.519–60.435)	190.47

^1^ Populations were reared on cotton and tested between 6 and 12 weeks after the original *B. tabaci* were brought from the field, when sufficient numbers of adults were available. The population code indicates its place in the order of populations collected from a given county. ^2^ Number of *B. tabaci* adults exposed to various concentrations of imidacloprid. ^3^ Concentration of insecticide active ingredient (mg. per liter). ^4^ LC_50_ relative to the susceptible (Laboratory) population.

**Table 2:** Dose response to thiamethoxam and resistance ratios of *Bemisia tabaci* adults, reared from Florida field-collected populations, as measured by mortality (LC_50_) with a cut cotton leaf and petiole systemic uptake bioassay in the laboratory.

Population Code ^1^	Mortality			Resistance Ratio ^4^
	No. Tested ^2^	Slope ± SE	LC_50_ (95% CL) ^3^	
Laboratory	506	0.564 ± 0.054	0.139 (0.021–0.460)	-
Hendry3	473	0.828 ± 0.058	0.965 (0.443–1.795)	6.94
Hendry2	384	0.705 ± 0.063	1.078 (0.388–2.288)	7.76
Manatee2	552	0.502 ± 0.031	1.228 (0.487–2.767)	8.84
Hillsborough3	450	0.522 ± 0.041	1.372 (0.420–3.418)	9.87
Manatee4	272	0.457 ± 0.047	1.492 (0.308–5.174)	10.73
Manatee3	581	0.620 ± 0.033	1.978 (0.916–4.011)	14.23
Hillsborough4	235	0.460 ± 0.045	2.027 (0.437–7.402)	14.58
IndianRiver-Emilia	278	0.570 ± 0.045	2.760 (0.797–8.254)	19.86
Manatee1	454	0.481 ± 0.040	3.057 (1.063–7.227)	21.99
IndianRiver-Squash	861	0.566 ± 0.026	3.621 (1.903–6.675)	26.05
IndianRiver1	382	0.707 ± 0.057	4.239 (1.947–8.458)	30.50
Hillsborough1	373	1.216 ± 0.072	4.477 (2.687–7.225)	32.21
Hillsborough5	227	0.477 ± 0.044	5.089 (1.592–15.549)	36.61
Hillsborough-Emilia	538	0.641 ± 0.032	5.636 (3.026–10.324)	40.55
Miami-Dade3	240	0.505 ± 0.045	10.076 (2.753–37.392)	72.49
Hillsborough2	470	0.821 ± 0.052	10.879 (5.734-21.010)	78.27
Miami-Dade1	577	0.812 ± 0.047	12.676 (7.853–20.769)	91.19
Hendry1	560	0.749 ± 0.043	16.484 (10.156–27.410)	118.59
Miami-Dade2	400	0.530 ± 0.052	24.430 (11.541–59.064)	175.76

^1^ Populations were reared on cotton and tested between 6 and 12 weeks after the original *B. tabaci* were brought from the field, when sufficient numbers of adults were available. The population code indicates its place in the order of populations collected from a given county. ^2^ Number of *B. tabaci* adults exposed to various concentrations of thiamethoxam. ^3^ Concentration of insecticide active ingredient (mg. per liter). ^4^ LC_50_ relative to the susceptible (Laboratory) population.

**Table 3:** Dose response to dinoteturan and resistance ratios of *Bemisia tabaci* adults, reared from Florida field-collected populations, as measured by mortality (LC_50_) with a cut cotton leaf and petiole systemic uptake bioassay in the laboratory.

Population Code ^1^	Mortality			Resistance Ratio ^4^
	No. Tested ^2^	Slope ± SE	LC_50_ (95% CL) ^3^	
Laboratory	499	0.564 ± 0.054	0.026 (0.002–0.122)	-
Manatee4	257	0.457 ± 0.047	0.043 (0.003–0.240)	1.65
IndianRiver-Emilia	298	0.570 ± 0.045	0.068 (0.009–0.289)	2.62
Hendry2	375	0.705 ± 0.063	0.125 (0.024–0.377)	4.81
Manatee2	515	0.502 ± 0.031	0.187 (0.056–0.497)	7.19
Manatee3	542	0.620 ± 0.033	0.189 (0.065–0.462)	7.27
Hendry3	467	0.828 ± 0.058	0.217 (0.075–0.494)	8.35
Hillsborough3	451	0.522 ± 0.041	0.320 (0.069–0.971)	12.31
IndianRiver1	363	0.707 ± 0.057	0.356 (0.099–0.891)	13.69
Hillsborough-Emilia	555	0.641 ± 0.032	0.396 (0.178–0.800)	15.23
IndianRiver-Squash	774	0.566 ± 0.026	0.494 (0.215–1.019)	19.00
Miami-Dade3	234	0.505 ± 0.045	0.556 (0.095–2.131)	21.39
Hillsborough2	431	0.821 ± 0.052	0.575 (0.200–1.298)	22.12
Hillsborough4	213	0.460 ± 0.045	0.587 (0.090–2.418)	22.58
Manatee1	462	0.481 ± 0.040	0.589 (0.149–1.621)	22.65
Miami-Dade2	361	0.530 ± 0.052	0.930 (0.284–2.228)	35.77
Hillsborough5	213	0.477 ± 0.044	0.953 (0.237–3.025)	36.65
Hillsborough1	330	1.216 ± 0.072	1.233 (0.624–2.196)	47.42
Miami-Dade1	539	0.812 ± 0.047	1.244 (0.642–2.175)	47.85
Hendry1	562	0.749 ± 0.043	3.350 (1.960–5.451)	128.85

^1^ Populations were reared on cotton and tested between 6 and 12 weeks after the original *B. tabaci* were brought from the field, when sufficient numbers of adults were available. The population code indicates its place in the order of populations collected from a given county. ^2^ Number of *B. tabaci* adults exposed to various concentrations of dinotefuran. ^3^ Concentration of insecticide active ingredient (mg. per liter). ^4^ LC_50_ relative to the susceptible (Laboratory) population.

**Table 4:** Dose response to flupyradifurone and resistance ratios of *Bemisia tabaci* adults, reared from Florida field-collected populations, as measured by mortality (LC_50_) with a cut cotton leaf and petiole systemic uptake bioassay in the laboratory.

Population Code ^1^	Mortality			Resistance Ratio ^4^
	No. Tested ^2^	Slope ± SE	LC_50_ (95% CL) ^3^	
Laboratory	491	0.564 ± 0.054	0.010 (0.001–0.060)	-
Manatee4	259	0.457 ± 0.047	0.011 (<0.001–0.085)	1.1
Hendry3	477	0.828 ± 0.058	0.117 (0.036–0.290)	11.7
Manatee3	574	0.620 ± 0.033	0.127 (0.041–0.323)	12.7
Hendry2	380	0.705 ± 0.063	0.140 (0.026–0.420)	14.0
IndianRiver-Emilia	287	0.570 ± 0.045	0.151 (0.026–0.566)	15.1
Manatee1	476	0.481 ± 0.040	0.177 (0.033–0.575)	17.7
IndianRiver-Squash	814	0.566 ± 0.026	0.189 (0.074–0.419)	18.9
Miami-Dade3	252	0.505 ± 0.045	0.193 (0.025–0.825)	19.3
Hillsborough3	439	0.522 ± 0.041	0.194 (0.037–0.633)	19.4
Manatee2	538	0.502 ± 0.031	0.269 (0.087–0.675)	26.9
Hillsborough4	251	0.460 ± 0.045	0.274 (0.041–1.094)	27.4
IndianRiver1	378	0.707 ± 0.057	0.325 (0.086–0.835)	32.5
Hillsborough2	541	0.821 ± 0.052	0.364 (0.121–0.823)	36.4
Hillsborough-Emilia	579	0.641 ± 0.032	0.379 (0.171–0.760)	37.9
Hillsborough1	337	1.216 ± 0.072	0.548 (0.250–1.041)	54.8
Miami-Dade1	558	0.812 ± 0.047	0.613 (0.289–1.136)	61.3
Hillsborough5	236	0.477 ± 0.044	0.646 (0.152–2.054)	64.6
Miami-Dade2	360	0.530 ± 0.052	0.790 (0.232–1.937)	79.0
Hendry1	585	0.749 ± 0.043	1.471 (0.802–2.477)	147.1

^1^ Populations were reared on cotton and tested between 6 and 12 weeks after the original *B. tabaci* were brought from the field, when sufficient numbers of adults were available. The population code indicates its place in the order of populations collected from a given county. ^2^ Number of *B. tabaci* adults exposed to various concentrations of flupyradifurone. ^3^ Concentration of insecticide active ingredient (mg. per liter). ^4^ LC_50_ relative to the susceptible (Laboratory) population.

## Abbreviations

The following abbreviations are used in this manuscript:

DNA: Deoxyribonucleic acid
GCREC: Gulf Coast Research and Education Center
LC: Lethal concentration
MEAM1: Middle East Asia Minor group of *Bemisia tabaci* (commonly known as biotype B)
MED: Mediterranean group of *Bemisia tabaci* (commonly known as biotype Q)
PCR: Polymerase chain reaction
TYLCV: Tomato yellow leaf curl virus
MDPI: Multidisciplinary Digital Publishing Institute
DOAJ: Directory of open access journals
TLA: Three letter acronym
LD: linear dichroism
